# Democracy and Sustainable Development

**DOI:** 10.1007/s44177-022-00019-z

**Published:** 2022-04-29

**Authors:** Dan Banik

**Affiliations:** Centre for Development and the Environment, University of Oslo, Oslo, Norway

**Keywords:** Democracy, Governance, 2030 Agenda, SDGs, China, India

## Abstract

The democratic discourse on climate change and sustainable development is becoming increasingly polarized. While some voters are pushing back against the movement to wean the world economy away from fossil fuels, others are questioning the huge costs that the transition to a green economy will impose and whether such attempts will have the required impact. This essay discusses the relationship between democracy and sustainable development by comparing the records of China and India in relation to the 2030 Agenda and the Sustainable Development Goals (SDGs). It argues that the politics of sustainable development has not received the attention it deserves. With a growing number of actors involved at various levels of society, the sustainable development narrative has often focused on a win–win narrative while glossing over areas where political agreement is more difficult to reach. While autocracies can achieve good results, democracy provides the best platform and guarantees for difficult negotiations and deliberations that are required for the achievement of sustainable development.

## Introduction

World leaders are being increasingly confronted with difficult decisions on development and the environment and the drastic cuts in greenhouse gas emissions that are required to address the climate crisis. Following growing awareness and anxiety among voters, environmental and climate issues have featured prominently in recent election campaigns in many parts of the world. Regular reports, including those published by the Intergovernmental Panel on Climate Change (IPCC), have generated considerable global media attention on the need for sustainable development and the bold decisions that are required to save the planet from overheating. Numerous social movements—such as the youth-led movement *Fridays for Future*, inspired by activists such as Greta Thunberg—are mobilizing new groups of citizens to protest and hold political and corporate leaders to account for inaction.

Underlying much of this activism is the concern about political decisions today that may radically impact planetary health and the lives of future generations. In retaliating against such movements and policies, opposition groups are mobilizing citizens against the drastic changes proposed by environmentalists and climate campaigners to current lifestyles and consumption patterns. The democratic discourse on climate change and sustainable development is thus becoming increasingly polarized. While some voters are pushing back against the movement to wean the world economy away from fossil fuels, others are questioning the huge costs that transitioning to the green economy will impose and whether such attempts will have the required impact.

What then is the relationship between democracy and sustainable development? In contrast to the voluminous literature on democracy and economic growth and democracy and poverty reduction, a search of the recent literature on the links between democracy and sustainable development reveals surprisingly little academic attention. Like many others before me, I argue that development is a contested notion and there is no one way of achieving it. Individuals and groups have differing opinions on how development, or the lack of it, affects them and what they believe is the correct way forward to achieve development. While climate change and global warming are indeed “global” issues, the discourse on sustainable development is unequally distributed in the world. Indeed, despite India, China and other major countries in the Global South showing growing interest on sustainable development, it is mainly the Western lens that is applied in many global debates and high-level forums and summits (Chakrabarty 2017). And while the concept of sustainable development has been rejuvenated following the adoption of the 2030 Agenda and the 17 Sustainable Development Goals (SDGs) by world leaders in 2015, not all debates have been settled and numerous disagreements persist on how exactly the global community ought to address challenges related to “Our Common Future”—the title of the Brundtland Commission’s influential 1987 report (WCED 1987). It is precisely because of the contested nature of “development” and the differential impacts it has on various groups on the population that we must focus greater attention on the politics of sustainable development.

Much of this article builds on research conducted in China and India since 2015 under the auspices of the Oslo SDG Initiative at the University of Oslo. The empirical material and perspectives on China draw from the project “CSR in the Sustainable Development Goal Era” (2015–2021). Together with colleagues at the University of International Business and Economics (UIBE) in Beijing, we studied sustainable development strategies and green entrepreneurship among small- and medium-sized enterprises (SMEs), start-ups, and state-owned enterprises (SOEs). The material on India is based on interactions with policymakers, journalists, scholars, students, and activists undertaken between 2018 and 2021, during which I also gave several talks on sustainable development at various Indian universities. I also participated at numerous seminars and conferences on sustainable development in China and India, which provided ample opportunities to interact with business leaders and managers, civil servants, environmental NGOs, think tanks, diplomats, UN officials, journalists, scholars, students, and civil society organizations. A final site for empirical material was the United Nations High Level Political Forum (HLPF) in New York, which I attended in 2018 and 2019. The HLPF provided ample opportunities to discuss, with politicians, diplomats, and civil society organizations not only the Voluntary National Reviews (VNRs) of the 2030 Agenda presented by UN member states but also progress in achieving the SDGs as well as the challenges faced by various countries, including China and India. Due to the politically sensitive nature of this research, all interviews have been anonymized.

The main argument in this essay is that the politics of sustainable development has not received the attention it deserves. With an increased number of actors involved at various levels of society, the sustainable development narrative has often focused on a win–win narrative while glossing over areas where political agreement is more difficult to reach. While autocracies can achieve good results, democracy provides the best platform and guarantees for difficult negotiations and deliberations that are required for the achievement of sustainable development. In part 1, I offer a brief overview of the two key concepts addressed in this article—sustainable development and democracy, reviewing some of the most relevant strands in the debate. Thereafter I examine, in part 2, the adoption of the 2030 Agenda and the Sustainable Development Goals (SDGs) in China and India and discuss the challenges and opportunities in terms of the democracy-sustainable development relationship in part 3.

## Part 1: Sustainable Development Ressurected

The concept of sustainable development gained considerable international attention following the Brundtland Commission’s 1987 report—*Our Common Future*. The definition of sustainable development—that has endured for almost three decades—entails fulfilling the needs of the current generation without compromising the ability of future generations to meet their needs (WCED 1987: 41). Two crucial aspects in this context are “the essential needs of the world’s poor, to which overriding priority should be given” and “the idea of limitations imposed by the state of technology and social organization on the environment's ability to meet present and future needs” (Ibid.). The popularity of the sustainable development concept has, however, ebbed and flowed for decades. In some parts of the world, such as the United States, the idea did not receive much attention and critics frequently highlighted the concept’s lack of analytical rigor (Victor 2006). Over the years, several scholars and policymakers have argued that since sustainable development is such an all-encompassing topic, it is very difficult to be against it, which in turn means it is difficult for politicians and administrators to prioritize specific initiatives over others. In a recent conversation with me, Gro Harlem Brundtland claimed that it has taken a relatively short period of time (just three decades) for the sustainable development concept to become mainstream, which is quite a feat for an idea that many considered radical and controversial in the late 1980s.^1^

Since the adoption of the 2030 Agenda by world leaders in 2015, the concept has not only been rejuvenated but also become an integral part of the global development narrative. Unlike the Millennium Development Goals (MDGs), which were limited in scope and applied only to low-income countries, the 2030 Agenda encompasses both development and environmental concerns and relate to the whole world. The SDGs have therefore been widely praised for being innovative in providing a broad and appealing framework to closely link “sustainability” with “development” through the principles of “universality”, “integration” and “leave no one behind”. Many scholars, activists and political leaders have thus claimed that the 2030 Agenda is transformative as it encourages a radical shift in world affairs by encouraging governments in both rich and poor countries to prioritize sustainable development that goes beyond simply focusing on economic growth to embracing a much broader agenda that promotes developmental and environmental concerns.

Despite the initial euphoria, however, progress on achieving the SDGs has been mixed (United Nations 2021). Apart from a few exceptions, governments all over the world have struggled to coordinate policies that promote both environmental and developmental outcomes. Most low-income country governments have been slow to allocate sufficient resources in domestic budgets for speedy implementation of the SDGs. Some of this is due to insufficient revenue generation while in other cases it is due to a general political reluctance to fully engage with the 2030 Agenda. Even the governments of more affluent countries have been slow to react, often making symbolic pledges without undertaking measures to make significant changes to existing policy. In still other cases, the private sector—large parts of which have warmly embraced the SDGs—have made grand declarations of their *intent* to engage with the SDGs but have not followed up with evidence of their actual actions and impact on the ground.

It is suffice to say that the SDGs have thus far not elicited the kind of enthusiasm among political leaders that is required for the success of such an ambitious agenda. In many countries, there is an on-going and often polarized national debate on the extent to which the country should be prioritizing “global goals” rather than goals that are more narrowly defined to apply to local situations (e.g., prioritizing allocation of resources to selected regions and targeting selected groups in the population) (Banik 2018). The 2030 Agenda appears to be at a crossroads. Although the world was off track before the emergence of COVID-19, the pandemic has reversed many development gains. Much of the progress in poverty reduction has been reversed and low-income countries are now worse off than before.

### What’s Democracy Got to Do with it?

It is often assumed that democracies possess the capacity to foster basic human freedoms, i.e., certain minimum freedoms relating to those of association, speech, expression, and opposition are essential for elections to be meaningful. The checks and balances inherent in a democracy prevent excessive abuse of power and arbitrary oppression while guaranteeing rights and entitlements to citizens and correcting policy errors. Diamond (1996: 21) distinguishes between minimalist conceptions of democracy and more substantial or substantive forms. Minimalist or “electoral democracy” includes “minimal levels of freedom in order for competition and participation to be meaningful” such as the ability to hold regular free and fair elections. Thus, democracy is a system “for arriving at political decisions in which individuals acquire the power to decide by means of a competitive struggle for the people’s vote” (Schumpeter 1947: 269). Others view it as “a political arrangement in which people select governments through elections and have a reasonable possibility of removing incumbent governments they do not like” (Przeworski 2019: 5). Accordingly, “democracy is simply a system in which incumbents lose elections and leave when they lose” (Ibid.).

A minimalist conception of democracy is often inadequate to tackle some of the challenges that people in many parts of the world face today. If we are to address not just poverty reduction but also many of the broader sets of challenges posed by climate disruption, we need strong and proactive state action that goes beyond just a focus on elections. Thus, at the other end of the spectrum lies the broad concept of “liberal democracy”, which includes periodic, free, and fair electoral contestation together with a system of checks and balances, the availability of alternative and multiple channels for expression and representation of views, and the right of being politically equal under the rule of law (Diamond 1996: 23–24). Between “minimalist” and “liberal” democracies, however, there are several intermediary versions of democracy.

The key issue here is the capacity of democracies, in comparison to non-democracies, to extend freedoms from those initially granted into more substantial ones. In consolidated and stable democracies with a well-developed political culture, “the range of rights and liberties available to citizens... goes well beyond what is strictly required for the existence of democracy itself” (Dahl 1992: 236). While this may often be the case, democracy and freedom do not enjoy a directly proportional relationship and maximizing both are a challenge for many societies. As Przeworski et al. (2000: 34) argue, ‘Whereas democracy is a system of political rights—these are definitional—it is not a system that necessarily furnishes the conditions for effective exercise of these rights.”

Some of the recent evidence on the state of democracy around the world paints a gloomy picture (Diamond 2019). For example, according to the Freedom in the World 2021 report, the coronavirus pandemic, economic uncertainty, and conflict have contributed to the general decline of global freedom (Freedom House 2021). Another report finds that with autocracies now home to 68% of the population, the world is currently in the “third wave of autocratisation” and electoral autocracies are the most common regime type (V-Dem Institute 2021). In the early 1990s, there was considerable optimism as many countries made the transition to democracy. The world today is, however, undergoing a democratic recession and certain countries such as India—that were long viewed to be the beacons of democracy—are now being termed as “electoral autocracies” or “partly free” (V-Dem 2021; Freedom House 2021). While there may be considerable disagreement over such classifications, there is widespread consensus among scholars that democracy is backsliding, which in turn is of major concern to those who extoll the virtues of civil and political rights and freedoms.

An important debate on development has been the role of democracy in promoting economic growth on the one hand and distributing the benefits of growth and reducing poverty on the other (Knutsen 2020). Elsewhere, I have operated with a definition of “development” —influenced by the work of Atul Kohli—where development is understood as a deliberate movement of societies towards a situation of more livable life conditions (Banik 2006, 2010). Three critical elements of such livable conditions are economic growth, some redistribution of growth, and democracy for the redistribution of the benefits of growth. Development is a process where these goals are to be maximized even though there may be trade-offs in the process. In terms of development within countries, the main challenge is an old challenge: how to secure rapid economic growth and distribute the benefits of this growth in a manner so that inequality is reduced. In this context, civil and political freedoms are crucial for the fulfilment of pressing economic need. Hence, the role of democracy in promoting development is crucial. For example, Knutsen (2020: 4) finds that democracies are better than autocracies in avoiding economic crises and are hence “a less risky proposition for citizens and investors alike”. Democracies also perform a safety-net function in that they guarantee avoidance of “the worst possible economic outcomes” (Ibid.).

Even though there is considerable democratic backsliding and democratic regression, the idea of democracy has many virtues (Diamond 2020). As Amartya Sen argues, the promotion and strengthening of a democracy is crucial for the process of development on three grounds (Sen 2000: 157–158). First, democracy has intrinsic importance in that it is a value in itself and has a direct relevance in the promotion of basic capabilities, which includes social and political participation. Second, democracy makes possible various instrumental contributions in ensuring that people can express and support their claims of economic needs and receive political attention. Third, democracy has a constructive role in the very understanding and identification of what constitutes ‘needs’ in a social context. Civil and political rights enable participation in public debates and discussions and allow criticism, which in turn is important for the understanding and conceptualization of economic needs, and the eventual response to meet such needs. Sen argues that each of these three features needs to be considered while evaluating a democratic system.

Democracy also allows citizens to make use of a range of opportunities available, but to what extent these are used will depend on the functioning of multiparty politics and the nature of dominant values in society (Sen 2000: 154–155). However, although democracies guarantee certain basic freedoms, there is no guarantee that citizens can adequately utilize these freedoms. As Beetham (1999: 91–92) observes, understanding democracy exclusively about a set of political institutions implies that institutions alone are all-important for democracy. This ignores the intrinsic role that human rights and freedoms play in the notion of democracy. Thus, how effective institutions are, i.e., how democratic they are, will depend on the guarantee and exercise of civil and political freedoms in society. And it is important to emphasize both the extent of freedoms guaranteed in a democracy and the “enabling conditions, institutional as well as social” (Przeworski et al. 2000: 34) that allow individuals to act freely in the political sphere—even though they may vary considerably from situation to situation.

Another set of issues relates to the ability of democracies to resolve or process conflicts. This may be achieved by designing political institutions in a manner that absorbs disagreements, which are thereafter channelized through institutions that have rules and regulations and predictability—facilitating a result that is acceptable to the parties of a conflict (Przeworski 2019)*.* Such ways of resolving democratic disagreements—whether it is a railway project that is harmful to wildlife, a coal-fired power plant that causes harmful environmental effects, or expensive subsidies to owners of electric vehicles—are often easier when the stakes are not too high or not too low. And individuals and groups that have lost out on a decision have the possibility of, and belief in, winning a favorable verdict at a later stage—for example, by helping to change the rules following new elections and a change in political circumstances.

One of the main criticisms has been that in democracies policies are often short-sighted, with politicians obsessed with being re-elected rather than looking for important long-term goals that may be politically unpopular. Consequently, the obsession with re-election influences politicians and their parties to adopt rhetorically appealing populist policies that are short-term oriented (the four or five-year cycle perspective) when longer-term perspectives are required. There is growing academic interest in understanding how democratic institutions can address these challenges. For example, Finnegan (2022) finds that electoral rules (e.g., proportional representation) and interest group intermediation are crucial for structuring the distributional politics of climate change. Thus, institutions may enable leaders to enact legislation that imposes short-term costs on voters. In *Why Nations Fail*, Acemoglu and Robinson (2012) argue that understanding why inefficient institutions persist is crucial. In the sequel to that book, they claim that the pursuit of liberty and development progresses along a narrow corridor (Acemoglu and Robinson 2019). The corridor is narrow because at one extreme are despotic states that repress people while on the other extreme there are absent states where violence and lawlessness rule. Liberty and prosperity therefore depend on a so-called “Shackled Leviathan”, which essentially entails two crucial factors: (a) a powerful state that can control violence and enforce laws while providing public services and (b) a strong mobilized society must control or put the shackles on the state, because checks and balances cannot be parachuted from above. In the sweet spot that is the narrow corridor, the state and society balance each other out (Ibid.).

In the ensuing sections, I examine the political interest in sustainable development and the adoption of the SDGs in two very different political systems—India and China. In Acemoglu and Robinson’s framework, China is a “Despotic Leviathan” with a weak society whereas India has underperformed on poverty reduction and development because the caste system and the “cage of norms” has shackled both state and society.

## Part 2: Adoption of the SDGs in China and India

The India–China comparison is particularly interesting in relation to the democracy and development relationship as the two countries appear to be, on paper, opposites given differing political systems. India is frequently referred to as the world’s largest democracy and has a mixed record on economic development. China is not democratic and has witnessed rapid economic growth and major improvements in human development in the past three decades. In terms of most indicators of development—such as infant mortality, primary health education, electricity coverage, infrastructure—China leads India. Several scholars have, over the years, argued that this impressive achievement is largely due to China’s investments in human development even before it began experiencing rapid economic growth (Drèze and Sen 1989). But Amartya Sen has also long argued the crucial role that democratic politics plays in India in preventing major disasters such as famines, which China was unable to prevent due to a lack of political freedom (Sen 1991, 1993). Indeed, since India achieved independence from colonial rule in 1947, it has not experienced a famine despite repeated situations of prolonged food insecurity and food crises. Sen and others have argued that this success in famine prevention in India has been possible because of freedom of speech and expression and the role that a free media and democratically elected politicians and their political parties play in national and local legislatures (Banik 2007). Thus, although parts of the country regularly experience periods of food insecurity, democracy has helped prevent mass starvation and a major and visible crisis such as famine. The Chinese experience has been somewhat different. Despite all its recent success, China experienced a major famine between 1959 and 1961/62 during the so-called “Great leap forward”, when an estimated 30–35 million people died. Several scholars, most notably Sen, have argued that it was the lack of democracy that created this human catastrophe. In the absence of a free media and independent political parties, the authorities were not held to account for the failure to mount an effective response to prevent starvation deaths, despite the availability of early warning information of impending mass starvation.

What about the track records of these countries in relation to sustainable development? Despite their widely varying records at preventing large and visible crises, both India and China have shown considerable political interest in, and support for, the SDGs. Since the adoption of the 2030 Agenda and the 17 SDGs by world leaders at the UN General Assembly in 2015, the leaders of China and India appear to have been active in supporting the operationalization of the SDGs in their national and local contexts. While the implementation of the SDGs has thus far received a mixed response from world leaders—and overall progress in achieving the goals during the past seven years has been slow—both New Delhi and Beijing appear to have warmly embraced these “global goals” and there is growing talk of the importance of sustainable development in administrative and political circles. Both countries have regularly submitted, at national and international forums, detailed reports documenting progress while also highlighting the numerous challenges they continue to face in achieving the SDGs. In addition, the Indian and Chinese governments have made a conscious attempt to develop national indicators for measuring progress on the SDGs and thereby operationalize these global goals into local realities.

### China and the SDGs

China has consistently argued that the SDGs must be “open and inclusive” and “transformative and innovative”. In various progress reports, Beijing has emphasized respect of national sovereignty and diversity in development models, and that all countries must enjoy equal rights to participation in international rulemaking. It played an active role in the process leading up to the 2030 Agenda and has long advocated the principle of “Common but differentiated responsibilities” according to which individual countries have different capabilities and differing responsibilities in addressing climate change. The State Council has the overall responsibility for SDG implementation within the country and is supported in this task by 45 members of the Inter-Ministerial Coordination Mechanism for China’s Implementation of the 2030 Agenda for Sustainable Development.

Soon after the adoption of the SDGs, the government published a National Plan on Implementation of the 2030 Agenda in 2016, which advocated strong support to the SDGs, and signaled the country’s keen interest in building on its impressive successes in relation to the Millennium Development Goals (MDGs) (Government of China 2016). The “guiding thoughts” for achieving sustainable development include an emphasis on development that is “innovation-driven”, “coordinated”, “green”, “open”, “shared” and “harmonious” (Ibid: 7–9). A subsequent “progress report” published in 2017 reiterated the country’s numerous achievements in registering “sound and steady growth”, improving the living conditions of its population and “all-round progress … in green development”. In particular, the report highlighted that “remarkable achievements have been made in deepening international development cooperation”, although it insisted, like most Chinese policy documents, that China remains the “largest developing country in the world” (Government of China 2017). In its 2019 progress report, Beijing highlighted numerous achievements while acknowledging challenges related to achieving greater equality in society. For example, it noted: “The gender awareness recognising equality between men and women is yet to prevail in China. Women’s status in the family and the level of social recognition given to domestic work are yet to be further improved. Due to constraints in resources, information and other factors, women in impoverished rural areas are still not sufficiently protected” (Government of China 2019: 27). The report also acknowledged the difficulty of balancing developmental goals with environmental goals.

In its latest Voluntary National Review (VNR), China highlighted its success in eradicating extreme poverty at the end of 2020, its commitment to tackling climate change and promote global green development, improvements in public health and its efforts to tackle the pandemic, its achievements in maintaining steady economic growth and its readiness to assume greater responsibility as “a major country” in promoting international development cooperation (Government of China 2021). Among the challenges ahead, the report listed the fight against COVID-19, the challenge of vaccinating a large population, maintaining sound economic and social development and its vision of building a global health community under the auspices of the United Nations (Ibid.: 45–46).

Since 2016, China’s progress reports and VNRs on the SDGs have highlighted a set of common issues. They typically emphasize the “great importance” and the “top priority” accorded to the implementation of the 2030 Agenda, which is said to be closely aligned with medium- and long-term national development strategies aimed at promoting balanced development that ensures economic, political, cultural, social and ecological progress. The approach adopted by China is described to be “people-centered” and one that builds on a “philosophy of innovative, coordinated, green, open and shared development” (Government of China 2021: 6). On the global stage, Beijing has consistently promoted the idea that the principles of the SDGs are closely linked to its signature foreign policy initiative – the Belt and Road Initiative (BRI). Thus, some UN officials and commentators have, for example, argued that the BRI’s five priority areas—policy coordination, facilities connectivity, trade and investment, financial integration, and people-to-people bond—are crucial for the promotion of the social, economic and environmental dimensions of sustainable development.^2^

China has also encouraged state-owned enterprises as well as private companies to closely integrate the SDGs into their business models, although it has mainly been large businesses that have thus far actively published detailed reports highlighting how their activities at home and abroad are linked with the SDGs (Banik and Lin 2019). In addition, Beijing has established new institutions to promote sustainable development such as the Center for International Knowledge on Development (CIKD), which is affiliated with the Development Research Center of the State Council (DRC). CIKD is mandated to pool Chinese and international resources to advance development studies, undertake research on development theories and practices, and promote research and knowledge-sharing on international development issues, including the implementation of the 2030 Agenda. Numerous other university institutes and thinktanks have also being established in the country—geared up to propagate a model of economic development that celebrates the Chinese experience, but with an added emphasis on sustainability as the need of the hour.

### India and the SDGs

The enthusiasm for the SDGs has been relatively high in India, at least at the central government level. Indeed, New Delhi believes that since India is “home to one-sixth of all humanity”, the country’s success in achieving the SDGs will have a global impact on the success of the 2030 Agenda (Government of India 2020: 3). The Indian government adopted a Three-Year Action Agenda early on (covering years 2017–18 to 2019–20) and there has been an attempt to adopt a “whole of society” approach on sustainable development by engaging a range of stakeholders including local and state governments, civil society organizations, local communities, and private sector actors. Prime Minister Narendra Modi has promoted the idea that India’s national development agenda should abide by the *Sabka Saath Sabka Vikas* (“Collective Effort, Inclusive Growth”) principle (Hall 2019). Like China, there is also strong support in the country for the idea of “common but differentiated responsibilities”. The task of coordinating and monitoring the implementation of the SDGs has been delegated to the National Institution for Transforming India (NITI Aayog) —a government think tank that replaced the erstwhile Planning Commission of India in 2015. It coordinates policymaking and facilitates regular interaction between central government ministries and state governments. NITI also organizes national SDG conclaves, where civil society organizations, scholars and civil servants are invited to reflect on progress and challenges and publishes annual SDG implementation reports.^3^ Further, NITI drafts India’s Voluntary National Review reports that are presented at national and international events, including the UN High Level Political Forum on Sustainable Development.

Interestingly, NITI has operationalized the global goals into locally compatible SDG indicators. It has created several indices to measure performance, including the School Education Quality Index, State Health Index, Composite Water Management Index, Sustainable Development Goals Index, India Innovation Index and Export Competitiveness Index. It regularly ranks Indian states on an SDG index, gently naming and shaming regional governments for their track record on the SDGs by grouping Indian states in four categories—aspirant, performer, front-runner, and achiever.^4^ With these rankings, NITI promotes so-called “competitive federalism” by encouraging inter-state competition and nudging states to improve their performance on sustainable development.

In numerous official progress reports on the SDGs, the Indian Government has claimed that at the macro level, the country’s policy focus and resource allocation for the main developmental programs are closely aligned with the SDGs. One of the programs that the government has highlighted in recent reports is *Ayushman Bharat*, which is the world’s largest health protection scheme, covering over 500 million individuals and is closely aligned with SDG 3 (health and well-being) and SDG 10 (reduced inequalities). Other programs that are similarly projected to showcase India’s embrace of the SDGs include *Poshan Abhiyaan* (National Nutrition Mission), *Aayushman Bharat* (National Health Protection Scheme) and *Beti Bachao Beti Padhao* (Care for the Girl Child). Another relevant initiative on sustainable development is the Aspirational Districts Program, which aims to fast-track development in 112 “relatively backward districts of the country” (NITI Aayog 2019: 4). While India does not have the financial resources that can match China’s BRI, it nonetheless aspires to increase its global influence. In this context, the government has actively promoted its climate action agenda by establishing the International Solar Alliance aimed at achieving the outcomes of SDG 7 (clean and affordable energy) although there is, thus far, little evidence of actual impact of this initiative.

Like China, India has achieved considerable success in poverty reduction. For example, it has “halved the incidence of multidimensional poverty by lifting 271 million from the most vulnerable sections of society out of poverty, while reducing extreme income poverty from 21.2 per cent in 2011 to 13.4 per cent in 2015” (Government of India 2020: 4). Despite such recent successes, large groups in the population continue to live in poverty. As such, a key policy focus for the government, according to India’s latest VNR presented at the United Nations in 2020, continues to be the war against poverty through initiatives that focus on improving nutrition, education, sanitation, health, and energy security. Similarly, new and established social protection programs typically aim to improve access to drinking water, electricity and housing, and basic services. A core feature of the 2030 Agenda is the “leave no one behind” principle, which according to India’s latest VNR “resonates deeply in the philosophy of Mahatma Gandhi and is also enshrined in our constitution” (Ibid.). As a result, a key focus area has been social inclusion policies that involve “legislative and executive action to create a level playing field, to universalise access to basic services and to address the challenges faced by communities in vulnerable situations in all spheres of life, such as nutrition, health, education, skilling and livelihoods, employment and social security” (Ibid.: 5). There has also been an emphasis on generating more jobs by improving agricultural infrastructure, productive assets, and entrepreneurship-based livelihood opportunities. In all these strategies, India promotes the idea that its success in achieving the SDGs will largely determine global outcomes.

India’s numerous and vibrant civil society organizations have played an important role in pressuring the central and state governments to adopt policies that are closely aligned with the 2030 Agenda. Indeed, they play a crucial role in holding the authorities to account for development failures. The role of the Indian parliament is also important in this context. For example, legislative oversight on the progress of the SDGs is undertaken by the parliament’s Public Accounts Committee, which conducts periodic reviews of the performance of NITI Aayog and related line ministries. In addition to making MPs aware of the details of the 2030 Agenda and the SDGs through briefing sessions and conferences, the parliament has convened several international events aimed at promoting SDG awareness in middle- and low-income country contexts (Government of India 2020: 32).

## Part 3: Discussion

At recent climate change conferences, such as COP 26 in Glasgow in October–November 2021, there has been considerable attention on the numerous challenges that China and India continue to face on the path to sustainable development.^5^ Among the most controversial decisions arrived at in Glasgow in November 2021, amidst powerful lobbying by China, India, and their allies, was that countries agreed to “phase down” rather than “phase out” coal. While rich countries in Europe and other developing countries, such as small island nations, met this single word change with dismay, according to India’s environment and climate minister, the revision reflected the “national circumstances of emerging economies”.^6^ However, despite considerable challenges that remain in both countries—the threats of climate change and large groups of the population that continue to live in poverty—both countries appear to have warmly embraced the SDGs. What explains this political interest in promoting sustainable development?

For decades, China has witnessed uneven development, with several central and western provinces lagging in most indicators of human development (Ravallion and Chen 2004). Similarly, there are regions in India that have historically lagged the rest of the country in relation to literacy, health, and overall human development (Chaudhuri and Ravallion 2006). While rural poverty in both countries remains high, there are also growing problems related to urban poverty and the overall quality of life for migrant groups who lack access to social services. The COVID-19 pandemic has made visible, particularly in India, the plight of migrants and other groups who rely on informal sector jobs for their livelihoods. Within China and perhaps less so in India, there is now a renewed debate about the costs of rapid economic growth. It thus makes perfect sense for both governments to exhort the virtues of sustainable development, particularly at a time when the public may be worried about a continued economic slowdown.

The rapid economic growth rates achieved by both countries in the past two decades has been much celebrated in academic and political circles. At the same time, there has been a growing demand in sections of society to revisit the basics of the current growth model amidst rising income inequalities. The model for social security in India, for instance, is mainly linked to formal jobs, and based on an employer–employee relationship. With the growth of the informal economy in the country, several civil society organizations and activists in India have pushed the government to adopt newer models of social security and an employment-centric growth model, as providing any type of work, leave alone decent work, continues to be a challenge for the Indian state. The unemployment challenge is particularly acute for women, a large majority of whom continue to work in the informal sector (agricultural work, childcare, caring for elders and the disabled, fetching firewood, etc.) but whose efforts are not counted as productive activity (Banik 2019; Chakraborty 2021).

Despite diverging political systems, both countries have adopted somewhat similar policies. Following the warm embrace of the SDGs by President Xi and Prime Minister Modi, China and India adopted national action plans and have tried to align their national development policies with the 2030 Agenda. Both countries have for decades designed and implemented some of the biggest and most ambitious social protection programs, many of which were relatively easy to align and re-brand with the 2030 Agenda. As such, neither country felt the pressure to introduce programs that were starkly different from those they pursued in a pre-SDG era. The nodal agencies in charge of coordinating and facilitating progress on the SDGs in both counties have operationalized the global indicators to suit their national, regional, and local contexts and issued regular reports documenting achievements and challenges in addition to listing areas for improvement. A common challenge for China and India is the quality of data available. Civil servants working for the central government in New Delhi as well as those working in various state capitals repeatedly mentioned the urgent need to improve the collection and analysis of good quality data that can measure SDG progress accurately at national and state levels. Such debates on data quality, are not common in China.

China and India also differ in significant ways. China’s economy is much larger than India’s, which in turn impacts SDG initiatives within their territories as well as SDG achievement abroad. Most importantly, democratic politics in India makes policymaking and implementation very different from what is commonplace in China. As mentioned earlier, India’s NITI has adopted a strategy of naming and shaming state governments for their track record on the SDGs. It thereby actively promotes “competitive federalism”—promoting and encouraging inter-state competition by shaming regional governments to improve their SDG performance. In India’s latest VNR, as in several other documents on the SDGs, the country’s 28 state and eight Union Territory governments are viewed as “pivots of localization”, particularly in areas where the Constitution empowers them to “play a leading role in determining the level of progress and prosperity of people under their jurisdiction while working in collaboration with the Central government” (Government of India 2020: 17). These include public health, education, agriculture, water, transport and communication, public order, and local government. Thus, the central government envisions playing an “enabling role” while state governments are expected to be “the key actors in the process of localisation of SDGs” (Ibid.). Despite the demarcation of powers between the centre and the states, federalism in India has been a contested concept since 1947. Indeed, centre-state relations have historically been frosty, especially when states are governed by a political party in opposition to the one forming the government in New Delhi. States typically complain of inadequate budgetary allocations while the centre accuses state governments of requesting additions grants without spending what has been previously allocated (Banik 2007). Moreover, democratic politics ensures that opposition to the policies of the central or a state government is not always based on merit but could be due to political affiliation. NITI prefers to adopt a more optimistic approach, claiming that the numerous challenges that India faces, such as those including health education, sanitation, and infrastructure, make the country “conducive for developing innovative solutions to address them and also provide a useful lens for solving similar problems in other parts of the world” (NITI Aayog 2019: 3).

Democracies also provide civil society with a powerful voice on sustainable development issues, especially on the challenges associated with a much-talked-about idea in the 2030 Agenda for sustainable development—“leave no one behind”. Ensuring inclusive development is a challenge for most countries, but more so for one that is as large and diverse as India. Among individuals and groups that are left behind in India are *Dalits* (ethnic groups considered to be at the bottom of the caste hierarchy), Adivasis (indigenous groups), migrants, construction workers, domestic helps, manual scavengers, refugees, survivors of violence, displaced groups, single women, the homeless, people with disabilities and widows. Some civil society organizations routinely question the extent to which the interests of these groups are currently represented in national forums and discussions. For example, the activist Suneeta Kar Dhar has questioned the wisdom of placing women and children in the same category in official data collection and reporting which, she argues, tends to “infantilise” women’s problems and needs (Banik 2019). Other organizations criticize the standard practice of treating the blind and deaf as passive “patients” that require benevolence and charity rather than viewing them as human beings and active agents shaping their own destinies. Still others claim that in the absence of regular dialogues, marginalized groups in India’s democracy are not just being “left behind” but actively “pushed behind”. Thus, despite democratic freedoms, scholars and activists argue that diverse groups in the population—women, people living with disabilities, transsexuals—ought to be better represented in debates, discussions, and policies (Ibid.).

### Pollution Control

Pollution control is an illustrative example of an area where both countries have achieved some success, although much work remains. It also exemplifies some of the opportunities and challenges of policy formulation and implementation in two very different political contexts. India and China have some of the most polluted cities in the world. Following a growing number of national and international media reports on air pollution in recent years, there has been heightened public scrutiny of official measures aimed at combating pollution in New Delhi and Beijing. According to AirVisual, a real-time air quality information platform by the Swiss air quality technology company IQ Air, the ten most polluted countries in 2020, weighted by population, were Bangladesh, Pakistan, India, Mongolia, Afghanistan, Omar, Qatar, Kirgizstan, Indonesia, and Bosnia Herzegovina (China is ranked 14th). South and East Asian cities were the most polluted globally in 2020 with Bangladesh, China, India, and Pakistan sharing 49 of the 50 of the most polluted cities worldwide (IQ Air 2020). A spate of media reports—building on IQ Air reports—have in recent years drawn attention to the fact that Beijing’s air has improved significantly in the past 5–7 years and the city has dropped out of the list of the world’s 200 most polluted cities. This is particularly impressive since Beijing was ranked the 52nd most polluted city in the world in 2016. Following media attention and increased concerns that were frequently and visibly expressed by Beijing’s inhabitants (many routinely wore masks while commuting to office and schools even before the onset of the COVID-19 pandemic), the Chinese government began paying greater attention to revising and enforcing various environmental regulations that had for long been gathering dust. The growing damage to Beijing’s international reputation caused by negative media reports on air pollution may have also played an important role in triggering political action. The authorities not only declared a “war on pollution” but also formulated ambitious environmental targets and began carrying out regular inspection campaigns to identify and impose heavy fines on violators. Polluting factories around Beijing were gradually shut down, the government imposed stringent restrictions on the burning of coal for heating, and heavy trucks were prevented from plying within the city. However, despite success in Beijing, many other Chinese cities continue to experience worsening air quality—a matter of great concern to citizens and policymakers alike. The key point is that while there is a growing amount of media attention on the perils of air pollution in China, there is little public debate within the country on the efficacy of the measures undertaken to combat the problem although several civil society organizations are increasingly active on pollution-related issues.

Like Beijing, New Delhi has also achieved some success and pollution has reportedly decreased by around 25% in the past four years. Following Beijing’s example, New Delhi has occasionally implemented a so-called “odd–even scheme” (first implemented in 2016) according to which vehicles—depending on whether the last digit in their license plate is even or odd – are allowed to drive on alternating days.^7^ The impact of this scheme in New Delhi is much debated in the country. One study found that although the scheme had limited impact on air pollution due to meteorological and crop-residue burning conditions, it considerably changed travel patterns and behavior of daily commuters in Delhi by increasing the car occupancy rates as well as usage of buses and the Delhi metro—which in turn had a major impact in reducing traffic congestion (Thakur and Qamar 2020). Others argue that what has helped to reduce pollution in and around the Delhi area is a clampdown on the burning of crop-residue in regions neighboring the country’s capital city, the closure of thermal power plants, the introduction of less-polluting forms of industrial fuel and the construction of highways that have bypassed the city.^8^

The role of democratic institutions has been crucial in the pollution control story in India. The Indian judiciary’s role is illustrative of such activity. For example, the National Green Tribunal (NGT) was petitioned in 2014 by the lawyer Vardhaman Kaushik. Based on this petition against rising levels of pollution, the Tribunal issued several orders that included a ban on old diesel and petrol vehicles. Firecrackers during the festive Diwali season have often increased pollution levels and the Tribunal has not only banned the sale and use of all firecrackers during Diwali but has consistently extended this ban over the years. For example, in December 2020, it declared: “There will be a total ban on the sale and use of all kinds of firecrackers during COVID-19 pandemic in the NCR and all cities/towns in the country where the ambient air quality falls under the ‘poor’ and above category”.^9^ Many of the orders by the NGT have subsequently been upheld by the Indian Supreme Court.

Unlike in China, where the causes of pollution are not critically discussed publicly, there is considerable debate and disagreement in Indian political, administrative, and academic circles on “which source contributes how much” to deteriorating air quality as well as the impact of pollution on health (Krishna and Ghosh 2021). While open public debate is crucial for sound policy decisions, some of the discussions are, however, not always guided by scientific evidence. For example, the Indian government has surprisingly claimed that there is inadequate evidence on the health impacts of pollution (Ibid.). Whenever the odd–even scheme is implemented in India, it results in public protests and considerable pushback from citizens and interest groups. While public protest against government action or inaction is a routine part of everyday life in democratic India, that is not necessarily the case in China. This does not mean that protests (or so-called “mass incidents”) do not take place in China. In a study of disputes on land acquisition and compensation in China (Zhou and Banik 2014), we found that protests against forced expropriation of farmland by local government authorities has been rising in recent years. We identified several institutional challenges that explained the growing phenomenon of social unrest over compensation, including the adjudication system itself, which remains an obstacle for farmers who wish to seek an administrative or legal remedy. The political, administrative, and judicial authorities in China prefer the maintenance of strict norms rather than facilitating more adaptable and feasible alternatives. The point is that such protests in China are usually addressed using legal rather than political tools. And while demonstrations against local government corruption or ineffective policies may on occasion be tolerated, public protests against central government policy are rare in China.

An important feature of the Indian political system is federalism, with the Indian constitution specifying the distribution of legislative, administrative, and executive powers between the central government based in New Delhi and state governments. Ever since India achieved independence from colonial rule, the Indian political system has witnessed recurrent center–state disputes and a political blame game, with state governments claiming that the center ignores states governed by opposition parties, while the center argues that states make unrealistic financial claims and do not utilize already available funds properly (Banik 2007). For example, the Government of Delhi (a union territory) is often at loggerheads with India’s central government. An illustrative example is that while the Delhi administration believes the odd–even scheme is effective for combating air pollution, the national transport ministry disagrees.

Several senior central government officials claimed in conversations with me that aligning the country’s national development agenda with the SDGs has not been as problematic as many would have imagined. They were of the view that since India has for decades implemented some of the most ambitious and costly social protection programs in the world, the country was able to make a smooth transition to the 2030 Agenda as several existing public policy initiatives were closely aligned with specific SDGs. However, other informants pointed to the discrepancy between the enthusiastic central government promotion of the SDGs and the lukewarm response to these from state governments. Managing center–state relations has always been challenging in the country’s federal set-up, and to motivate state governments to show greater ownership for the 2030 Agenda, NITI adopted a competitive federalism approach—gently naming and shaming non-performers. However, the general public is not necessarily more aware of environmental and/or climate change-related challenges due to such activity on the part of NITI. My general conclusion is that much of the focus on SDG implementation initiated by the central government is concentrated at the civil servant level. Unless citizens are mobilized and persuaded to make a greater contribution, sustainable development will mainly be viewed as an administrative exercise.

There are also differences in opinion within the Indian government on the potential impact of India’s ambitious policy on electric vehicles to combat air pollution in urban areas. In July 2019, the central government proposed that India will have only electric three-wheelers (“autorickshaws”) by 2023 and electric two-wheelers (scooters, motorcycles) by 2025.^10^ While many have lauded the push for electric vehicles aimed at reducing pollution, sceptics point to India’s continued reliance on highly polluting coal-powered plants for its electricity.

## Concluding Remarks

The idea that “no goal should be met unless it is met for everyone” is now well-established in the rhetoric around sustainable development and the SDGs and entails ensuring that every individual all over the world achieves the entire range of rights and opportunities articulated in the 2030 Agenda. However, what this means in practice, in various national contexts, remains unclear. Some influential actors (e.g., big businesses) that have embraced the idea of sustainable development in recent years, often give the impression that the world has finally found a magic bullet and that if only all countries pursued the roadmap identified in the 2030 Agenda, sustainable development will become a reality. I have argued that this is not necessarily the case. The sustainable development concept, and its application, remains highly contested, which in turn requires increased deliberation. Rather than becoming complacent, states and societies must continuously deliberate the scientific evidence available, the extent to which society demands change and the willingness of political actors to undertake bold initiatives. While democracy is necessary and often considered to be the best system that guarantees such debate and discussion—through negotiations, bargaining, strikes, and voting behavior—it is not a sufficient condition to ensure sustainable outcomes. The extent to which so-called “agents of change” such as youth activists communicate and address institutional weaknesses and ethical dilemmas (and the response from dissenting societal groups), is going to be crucial going forward. Other challenges relate to a rapidly growing population, inter-generational conflicts, and growing consumption, and how these issues are resolved in countries, such as China and India.

A key argument in support for democracy is, of course, the primacy of political rights in the articulation of economic, social, cultural, and ecological needs and desires. Without the freedom of expression, and alternative channels of information, pursuing any form of development is difficult. And while authoritarian leaders may be able design and implement effective policies to deliver development, these are most likely not based on genuine consultations with citizens and recipients of development interventions. Democracies guarantee that citizens can articulate an alternative vision of development, which may often differ in substantial ways from the visions of their leaders. There is a growing amount of literature that argues that democracies are better able than non-democracies to mobilize considerable public action when harm is imminent and visible rather than when threats are more long-term and currently less visible. However, democracies also have several perceived shortcomings, including short-terminism as politicians are driven by the hope and expectation of winning re-election when they may choose to pursue popular rather than effective policies. The key challenge that must be addressed relates to the ability of societies to adopt a long enough perspective that nonetheless receives short-term acceptance in the electorate (one example would be to have a 10-year perspective to make the transition from fossil fuel to electric vehicles as has been the case in Norway).

Another set of issues relates to the role of key institutions in democracies and non-democracies in upholding and promoting the tenets of sustainable development. The role of an independent judiciary, able to issue politically unpopular judgements is crucial in this regard. When citizens, civil society organizations and political groups petition the courts, they must believe that the judges will adopt an impartial assessment of the evidence presented. Even in a democracy such as India, there are growing concerns that sections of the judiciary are politically biased in favor of the ruling party. Even when the courts, based on popular citizen protests and movements, issue controversial but far-sighted decisions, the extent to which these are bypassed by politicians or selectively enforced often determine a society’s success in achieving sustainable development.

The discussion on Chinese and Indian strategies on the implementation of the SDGs illustrates the importance of what I believe ought to be a prioritized research agenda on global development going forward—how new policies on sustainable development create a new politics. While there is a considerable amount of literature on policy feedback (Skocpol 1992; Pierson 1993), we need greater attention on examining changes in preferences of social and political actors and broad patterns of civic participation on how climate and sustainable development policies as packages of resources affect interest groups, state capacities and mass publics and how new sources of information affect patterns of cognition, understanding and meaning. A related issue is how policies promoting sustainable development have the potential of transforming institutions and state capacity, which in turn will affect administrative capacity to undertake policy in the future. Politicians are typically consumed by the pressure to resolve numerous current problems and challenges and may not thus find it politically beneficial to engage in discussions regarding *future* problems that could affect a generation that is yet to be born. Similarly, many global policy recommendations often overlook issues of local justice and messy local political realities including competition between groups for control over scarce resources. Thus, the goal of promoting sustainable development today with an eye on the well-being of future generations appears illusory for governments struggling to solve current problems of poverty and deprivation within their borders. We require a renewed focus on examining the *process* by which globally negotiated policies are adopted in national forums, and the post-adoption effects of these policies.

A final point relates to the role of communication, which is often a challenge in autocracies but also increasingly a challenge in democracies (Runciman 2018). Much of the discourse on climate change in recent years has focused on doom and gloom scenarios of impending catastrophes. While there is indeed good reason to be concerned about the lack of progress in adapting to climate change, an excessive focus on doom and gloom narratives by political leaders and activists of climate change effects may result in inaction. The extent to which societies can use communication strategies that are rooted in persuasion rather than force or coercion is going to be crucial in all political systems.

## Footnotes

1 “Gro Harlem Brundtland on sustainable development, global responses to COVID-19 and the role of the WHO”, *In Pursuit of Development* podcast, 26 January 2021, https://in-pursuit-of-development.simplecast.com/episodes/gro-harlem-brundtland.

2 “Integrated policy approaches to the SDGs”, United Nations Department of Social and Economic Affairs (UN-DESA), https://www.un.org/development/desa/dpad/2017/integrated-policy-approaches-to-the-sdgs/.

3 I attended the second national conclave co-organized by NITI and the National Foundation for India in New Delhi in February 2019 and interacted extensively with civil society organisations and civil servants representing Indian states that presented their annual reports.

4 See https://sdgindiaindex.niti.gov.in/.

5 “After COP26, what’s the way forward for India”, *The Indian Express*, 03 December 2021, https://indianexpress.com/article/cities/pune/after-cop26-whats-the-way-forward-for-india-7653359/; “China at COP26: Coal, 1.5C and short-term actions”, *China Dialogue*, https://chinadialogue.net/en/climate/coal-1-5c-and-short-term-actions-china-at-cop26/.

6 “U.N. climate agreement clinched after late drama over coal”, https://www.reuters.com/business/cop/un-climate-negotiators-go-into-overtime-save-15-celsius-goal-2021-11-13/.

7 “Delhi to have odd–even scheme despite a dip in pollution”, https://www.livemint.com/news/india/delhi-to-have-odd-even-scheme-despite-a-dip-in-pollution-1568621145884.html.

8 “Explained: Air pollution in Delhi drops 25% in four years—what worked?”, *The Indian Express*, 13 September 2019,https://indianexpress.com/article/explained/how-delhi-cleared-the-air-pollution-air-quality-index-arvind-kejriwal-5987651/.

9 National Green Tribunal extends ban on firecrackers, The Hindustan Times, 03 December 2020, https://www.hindustantimes.com/delhi-news/national-green-tribunal-extends-ban-on-firecrackers/story-zkgiEHNozKcMRq3JWqJWJL.html.

10 “India turns to electric vehicles to beat pollution”, *BBC News*, 24 July 2019, https://www.bbc.com/news/world-asia-india-48961525.
